# Challenges of Cooperation between the Pre-hospital and In-hospital Emergency services in the handover of victims of road traffic accidents: A Qualitative Study

**DOI:** 10.17533/udea.iee.v37n1e08

**Published:** 2019-01-06

**Authors:** Hasan Jamshidi, Reza Khani Jazani, Ahmad Alibabaei, Shahram Alamdari, Majid Najafi Kalyani

**Affiliations:** 1 Shahid Beheshti University of Medical Sciences, Tehran, Iran. Shahid Beheshti University of Medical Sciences Shahid Beheshti University of Medical Sciences Iran; 2 Shahid Beheshti University of Medical Sciences, Tehran, Iran. email: r.khanijazani@sbmu.ac.ir. Corresponding author. Shahid Beheshti University of Medical Sciences Shahid Beheshti University of Medical Sciences Iran r.khanijazani@sbmu.ac.ir; 3 Shahid Beheshti University of Medical Sciences, Tehran, Iran. email: ahmadalibabaee@gmail.com Shahid Beheshti University of Medical Sciences Shahid Beheshti University of Medical Sciences Iran ahmadalibabaee@gmail.com; 4 Shahid Beheshti University of Medical Sciences, Tehran, Iran. email: alamdsari@endocrine.ac.ir Shahid Beheshti University of Medical Sciences Shahid Beheshti University of Medical Sciences Iran alamdsari@endocrine.ac.ir; 5 Nurse, Ph.D. Assistant Professor, Shiraz University of Medical Sciences, Shiraz, Iran. email: najafikalyani@sums.ac.ir Shiraz University of Medical Sciences Iran najafikalyani@sums.ac.ir

**Keywords:** ambulances, accidents, traffic, patient handoff, personnel, hospital, health resources, emergency service, hospital resources, qualitative research, ambulancias, accidentes de tránsito, pase de guardia, personal de hospital, recursos en salud, servicio de urgencia en hospital, investigación cualitativa, ambulancias, acidentes de trânsito, transferência da responsabilidade pelo paciente, recursos humanos em hospital, recursos, serviço hospitalar de emergência em saúde, pesquisa qualitativa

## Abstract

**Objective.:**

To take a deep look at the challenges of cooperation between the pre-hospital and in-hospital emergency services in the handover of victims of road traffic accidents.

**Methods.:**

This is a qualitative study and the method used is of content analysis type. Semi-structured interviews were used to collect the data. Through purposive sampling, fifteen employees from ambulance personnel and hospital emergency staff were selected and interviewed. They expressed their experiences of cooperation between these two teams in the handover of traffic accident casualties. The interviews were transcribed verbatim and content analysis method was used to explain and interpret the content of the interviews.

**Results.:**

Three major categories were derived from the analysis of interviews: *Shortage of infrastructure resources* (Shortage of equipment, Shortage of physical space, and Shortage of manpower); *Inefficient and unscientific management* (Shaky accountability, Out-of-date information based activities, Poor motivation, and Manpower low productivity); and *Non-common language* (Difference in understanding and empathy, and Difference in training and experience).

**Conclusion.:**

The obtained results of this study suggest that the careful planning of resources, the promotion of managerial practices as well as empowerment program of the staff, healthcare managers and policymakers can take a pace forward in order to enter into a hearty coordination between these two services for the attention of victims of road traffic accidents.

## Resumen

**Objetivo.:**

Examinar los desafíos para la cooperación entre los servicios de emergencias prehospitalarias y hospitalarias **en** la entrega de víctimas de accidentes de tránsito.

**Métodos.:**

Estudio cualitativo con análisis de contenido. Se utilizaron entrevistas semiestructuradas para la recopilación de los datos. Se seleccionaron y entrevistaron quince personas de los equipos de atención prehospitalaria y del servicio de emergencias de un hospital a partir del muestreo intencional. Los participantes expresaron sus experiencias de cooperación entre estos dos grupos en la entrega de víctimas de accidentes de tráfico. Las entrevistas se transcribieron textualmente y se utilizó el análisis de contenido para explicar e interpretar el contenido de las entrevistas.

**Resultados.:**

Emergieron tres categorías principales*: Escasez de recursos de infraestructura* (equipos, espacio físico y de personal); *Gestión ineficiente y no científica* (responsabilidad inestable, actividades basadas en información desactualizada, motivación deficiente, y baja productividad de personal); y *Lenguaje no común* (diferencia en comprensión y empatía, y diferencia en capacitación y experiencia).

**Conclusión.:**

Los resultados obtenidos en este estudio muestran dificultades con la planificación de los recursos y la promoción de las prácticas de gestión, así como la necesidad de un programa de empoderamiento del personal. Se deben formular políticas para poder avanzar en el mejoramiento de la coordinación de los servicios en la atención a víctimas de accidentes de tráfico.

## Resumo

**Objetivo.:**

Examinar os desafios para a cooperação entre os serviços de emergências pré-hospitalar e hospitalar **na** entrega de vítimas de acidentes de trânsito.

**Métodos.:**

Estudo qualitativo com análise de conteúdo. Se utilizaram entrevistas semiestruturadas para a recopilação dos dados. Através da amostra intencional se selecionaram e entrevistaram quinze pessoas das equipes de atenção pré-hospitalar e do serviço de emergências de um hospital. Os participantes expressaram suas experiências de cooperação entre estes dois grupos na entrega de vítimas de acidentes de trânsito. As entrevistas se transcreveram textualmente e se utilizou a análise de conteúdo para explicar e interpretar o conteúdo das entrevistas.

**Resultados.:**

Emergiram três categorias principais*: Escassez de recursos de infraestrutura* (equipamentos, espaço físico e de pessoal); *Gestão ineficiente e não científica* (responsabilidade instável, atividades baseadas em informação desatualizada, motivação deficiente, e baixa produtividade de pessoal); e *Linguagem não comum* (diferença em compreensão e empatia, e diferença em capacitação e experiência).

**Conclusão.:**

Os resultados obtidos neste estudo mostram dificuldades com a planificação dos recursos e a promoção das práticas de gestão, assim como a necessidade de um programa de empoderamento do pessoal. Se devem formular políticas para poder avançar no melhoramento da coordenação dos serviços na atenção as vítimas de acidentes de trânsito.

## Introduction

Road traffic crashes, as one of the biggest public health problems, are of man-made crises which cut short the lives of approximately 1.2 million annually and leave between 20 and 50 million people injured and disabled worldwide.(1) Traffic accidents are the second leading cause of death in Iran. Iran is one of the countries with the highest rate of fatalities from road traffic injuries worldwide.(2) Traffic crashes kill about 28 000 people and leave 300 000 people disabled and cost Iran's economy billions of dollars. Road traffic injuries are the leading cause of death among people under 40 and the second leading cause of death in all age groups.(3) Every year, millions of people are hospitalized for a long time due to road traffic injuries who may not be able to return to their normal life, work, or their previous role in society.(4) Pre-hospital emergency is an important link between managing emergency medical response to victims off the hospital and their treatment in the hospital.(1) One of the vital wards of any hospital is emergency department in which the patients are handed over to the nurses in health facilities by the emergency medical services (EMS) staff before they are visited by a physician.(5) Hearty cooperation between the staff of these two units while handing over the road traffic casualties is a significant and critical factor affecting the process of their health.(6) 

Poor cooperation between pre-hospital and in-hospital emergency is considered as one of the main causes of high mortality rate of road traffic accidents in the developing countries and one of the biggest preventable challenges.(7) The complexity and unpredictability of working in hospital emergency departments, professional problems, time constraints, huge crowd of referrals, noisy and stressful environment at the time of the patient handover lead to missing of patients' information, exchanging false information, causing multiple errors and exposing cooperation with special challenges.(6,8) Despite the problems of cooperation between pre-hospital and in-hospital emergency in the handover of road traffic casualties, which is one of the main reasons for an increase in the mortality rate of traffic casualties, a few studies have been conducted on the challenges of collaboration in the handover of such patients which are mainly quantitative ones.(6,8,9) Since the cooperation between the pre-hospital and in-hospital emergency is influenced by such social, cultural and value factors whose identification requires a deep understanding of how cooperation is attained, it is clear that the data collected by quantitative studies using one or more questionnaires containing some objective and close-ended questions will not be able to show all aspects of this phenomenon in Iran. Moreover, most of the questionnaires used in quantitative studies have been prepared by researchers from other countries and they are mainly based on the concepts extracted from qualitative studies conducted in the same countries which of course conform to cultural, value, social and professional standards of those countries. There are lots of issues on how pre-hospital and in-hospital emergency can enter into cooperation which are still unsolved or require further investigation in their cultural, social, value, and professional contexts. Accordingly, considering the high rate of road traffic accidents inflicting enormous financial losses and mortalities in Iran, the researchers prompted this qualitative study to deeply understand the existing challenges in the cooperation between the pre-hospital and in-hospital emergency departments and make use of the findings in health care management.

## Methods

This study is a qualitative one and the method used is of conventional content analysis type. This method was chosen because it is an appropriate way to extract valid and reliable findings from text data. It creates new ideas, knowledge and facts and can be used as a performance practical guide. Through compressing and extensive description of the phenomenon in this method, the ultimate goal which is extraction of concepts and descriptive categories can be achieved. Formation of the concepts and categories serves to build a model, a conceptual framework, a concept map or categories.(10) As the research environment of qualitative studies must be realistic and natural, the present study was carried out in pre-hospital and in-hospital emergency centers as natural settings. The research community was employees of these two medical settings. The inclusion criteria for participants were: a) having professional work experience for at least one year in pre-hospital or in-hospital emergency; b) willingness to recount their experiences related to cooperation between pre-hospital and in-hospital emergency in the handover of traffic road injured patients. Purposive sampling began in 1394 and continued with theoretical sampling until data saturation. The samples were chosen from the employees of pre-hospital and in-hospital emergency departments with whom the researchers had in-depth interviews individually. 

To collect the data, the researchers used semi-structured interviews, and in-field notes. All face-to-face interviews began with asking an open question such as "Could you talk about your routine daily work?" and to clarify the issue further, such guiding questions as "Could you make a specific example?" or "In case of a problem, what would you do"? The objectives of the research were adjusted on the participants' responses and follow-up study questions were raised for elaboration of the concept under study. Questions for future interviews were based on the categories emerged. The interviews lasted 55 minutes, on the average. At first, the objectives of the research, the methodology of the interview, the participants' free will to take part or withdraw from the study were explained to them. Furthermore, the participants' permission was asked to record the interviews. In the meantime, a written informed consent was obtained from every one of them. Initially, the content of the interviews was recorded and then they were set down word by word by the researcher. In order to get a gist of the data gathered, the researcher reviewed them simultaneously a few times. Through conventional content analysis, meaning units were identified out of the words, sentences and paragraphs in the interview texts on "Challenges of Cooperation between Pre-hospital and In-hospital Emergency in the Handover of Traffic Casualties". After the manifest and latent concepts based on the participants' description were identified, the concepts and the codes were outlined. Then, the codes and the concepts were classified with their similarities and differences. Finally, based on continuous thinking, interpretation and constant comparison of data, the categories and key concepts underlying the data were extracted. Having outlined the concepts and the codes, the researchers extracted the themes. 

The data were then analyzed using the constant comparative analysis methods and inductive content analysis method. To achieve trustworthiness (Credibility) of the data, the researchers used protracted involvement, integration in data collection, frequent review, revision supervisor and constant comparison. To achieve dependability reflecting the reliability and stability of the data, member check was used in the form of peer views, and reviewing the comments written by the participants. Conformability of the data was achieved by submitting the reports, the comments and the notes to two relevant professors and winning their approval. Transferability of the data was ensured by rich description of the data.(11,12) The principles of confidentiality, obtaining written informed consent to participate in the interview and record the conversation, having the right to withdraw from the study at any time were among ethical considerations which were observed while carrying out this study. The study was approved by the Ethics Committee of Shahid Beheshti University of Medical Sciences, Tehran, Iran.

## Results

The participants in this study were 15 employees at pre-hospital and in-hospital emergency departments who were purposefully selected as samples. They all had rich experience and they were willing to participate in the study. The average age of the participants was 35 and they had 8 years’ professional work experience. The themes of the gathered data from the participants' responses were classified into main and sub-categories. The major categories included insufficient infrastructure resources, inefficient and unscientific management and non-common language. (Table 1.

**Table 1:** Major categories and subcategories derived from the data

*Major categories*	*Subcategories*
*Shortage of infrastructure resources*	Shortage of equipment
	Shortage of physical space
	Shortage of manpower
*Inefficient and unscientific management*	Shaky accountability
	Out-of-date information based activities
	Poor motivation
*Non-common language*	Manpower low productivity
	Difference in understanding and empathy
	Difference in training and experience

### Insufficient infrastructure resources

In view of the participants, one of the barriers in the way of collaboration is inadequate infrastructure resources. Lack of inadequate infrastructure resources would cause ambulance and patient delay at the time of handover, and tension among staff, and influence these two important parts of the health system. The subcategory of this category includes: shortage of equipment, physical working space, and manpower which are dealt with below.

### Shortage of equipment.

One of the themes obtained was that of shortage of the equipment. It was considered as one of significant challenging factors in entering into a hearty cooperation between pre-hospital and in-hospital emergency personnel by the participants. Unless shortage of equipment is met, attainment of such cooperation is not feasible. Faced with shortage of equipment, the personnel had different experiences in dealing with like showing patience and endurance, and linger in order to prevent tension and conflict. Some of the participants had made an attempt to solve the problem by reflecting the issue to the highest in authority; however, they were not satisfied with the outcome of their efforts. Participant No. 1 said: *When we reach the hospitals, there is no stretcher. We ask the triage personnel why there is no stretcher. They answer, Stretchers are few. Now you have to wait until they arrive*. Sometimes, they get in touch with the hospital supervisor to follow up. In fact, the supervisor does, but by the time the supervisor has it brought from another ward, from the 1^st^, the 2^nd^, or from the 3^rd^ floor or somewhere else, time will have passed. It is some nerve-racking between me and the hospital personnel. The same is true with triage personnel. We, the two parties, cannot deal with the patient at ease of mind. In the end, it takes half an hour, forty-five minutes or sometimes even an hour to fetch a stretcher and move the patient. Participant No. 8 said: *Done. After handing over the patient, I myself lingered for 10 minutes to take delivery of the devices, for example, taking delivery of a long backboard used for the patient, and that I must do*. Another participant said: *The next problem is lack of stretchers, backboards and scopes, so patients are delayed and handover cannot be done*. Many of the participants in the study believed that shortage of equipment is one of the barriers in the way of cooperation between pre-hospital and in-hospital emergency departments and leads to a waste of time and confusion of pre-hospital emergency personnel.

### Shortage of physical working space.

The other cooperation challenge experienced by most of the personnel was shortage of physical working space for the prompt handover of the patients to the in-hospital emergency ward and return to the emergency base. Shortage of physical working space affected timely and scientific handover of the patients and sometimes the continuity of treatment as well. Participant No. 2 said: *The capacity of the hospital emergency ward is limited. Imagine, the ward has 70 beds and stretchers for 70 patients. If it became 71 patients, it would mean a problem. We'll go there, we'll see everywhere is crowded with patients hospitalized even in the corridors. We have difficulty fighting our way through the crowd, let alone handing the patient over*. The other participant said: When there are lots of car crashes or in the case of special occasions like Nowruz Eid (The first day of a new year in Iran), there is not enough room for such patient volume in the hospital emergency ward. Participant No. 5 from hospital staff said: *Triage has little space for patients*. In addition to the shortage of equipment, shortage of physical space for patients' admissions is an important reason in the delay of pre-hospital emergency technicians. In some cases, shortage of physical space has interfered with the handover of the patients.

### Shortage of manpower.

 Manpower shortage is also one of the challenges on the way of attainment of cooperation between pre-hospital and in-hospital emergency departments in the handover of traffic casualties on which the participants focused on. Participant No. 3 from pre-hospital emergency staff said: *There are more than 50-60 patients in the emergency ward with 7-8 nurses, each of whom is to receive about 10 patients and that very nurse has to deal with their problems from A to Z. Nothing is done for the patient. Things like line, filing, visiting the patient, implementing attending physician's orders. The workload in emergency ward is three times as much as other wards*. *In a case where a heavy accident had happened, all the nurses were busy dealing with their patients. There was no free nurse to be able to take delivery of the new victims and attend to their needs*, No. 2 interviewee said. Another participant from pre-hospital emergency ward said: *Shortage of manpower has made us feel tired and this sort of fatigue can have an adverse effect on our cooperation*. The participants believed that shortage of manpower is a factor that causes wear-and-tear of the staff's physical bodies, a disruption in the handover of the patients and continuity of their treatment, and leaves emergency technicians with lots of delay in the hospital.

### Inefficient and unscientific management

One of the obtained themes was that of inefficient management. In participants' point of view, poor and inefficient management was considered as one of the most important challenges in entering into a hearty collaboration between pre-hospital and in-hospital emergencies. They stated that not paying attention to scientific and methodological management in these two departments would cause a lot of problems. This main category has four sub-categories: shaky accountability, doing activities on out-of-date basis, inadequate motivation, and low productivity of manpower, described as follows:

### Shaky accountability.

Most of the participants considered the low level of accountability of staff and authorities of both pre-hospital and in-hospital emergencies responsible for lack of coordination. This poor accountability made the nurses take the delivery of the patients from the pre-hospital emergency improperly, followed by creation of some problems for pre-hospital emergency staff as well as the patients / injured patients. Along with this line of argument, participant No. 13 said: *In case of accidents which are critical conditions and there are lots of casualties, despite the fact that hospitals are informed of such a condition by the Direction Headquarter, the hospital does not go through phases of crisis management to get ready for admission of the injured people*. Participant No. 6 said: *You do the handover of the patient to one of the staff. Someone else will arrive and says, I'm in charge of triage*. *So, you must repeat the handover process and hand in the report to the one claiming as responsible. Another scenario is that you already did the handover of the patient and have left the hospital. After a while, a call rings saying that you have not done the handover of the patient to the triage*. Participant No.2 said: *What are we going to do with the patient carried here? They say, Go and deliver to the triage. We answer, They're busy now. They would reply, it is nothing to do with us. So, we don't know what to do.* The participants stated that this very poor and shaky accountability, in addition to causing confusion and linger of the staff, not only causes dissatisfaction of the patients and those accompanying by but it will also affect the health outcomes of the patients.

### Doing activities on out-of-date basis.

One of the challenges of cooperation was doing activities on out-of-date basis so that it showed the personnel's scientific information and practical activities not be updated. Therefore, some of the therapeutic measures taken for the patient were not considered as acceptable to the other party. Despite having new scientific information, some of the emergency personnel avoided applying them to the patients in order to experience less challenges at the time of handover. Along with this line of argument, participant No. 3 said: *According to the 2015 version of CPR, the most important task for a patient in need of CPR is to perform a cardiac massage within the very first few minutes. Identification of vein and chip intubation is not very important. On carrying the patient to the hospital, I get into discussion with the nurse.* The nurse asks such questions as: *Why aren't your patient with a line? Why didn't not you get the patient intubated? If not doing the procedures demanded by the nurse may not have some bad consequences to me this time, I'll put my effort into the things they demand next time.* Say, *if they pick on me next time, I'll carry the patient intubated. This means that I am forced to do an unscientific task. Sure, this will count against the patient. Spending one more minute, I'll get the patient lined and intubated not to be picked at by the hospital nurses next time*. Another participant said: *Some things have become routine somehow and everyone thinks he is doing the 'right thing'. If someone wants to do his job to the standard, he is being told What are you doing? Let it go. .... After a while, you will get discouraged, and you'll do as the others do*. Doing daily routine work and lack of paying attention to the scientific principles of taking care of patients / casualties is one of the challenges faced by pre-hospital and in-hospital emergency personnel which leads to conflicts during the handover of patients.

### Inadequate motivation.

In participants' views, not paying attention to spiritual and material motivation was one of the challenging factors in attainment of collaboration that overwhelmed their willingness to work and overshadowed their interaction with colleagues. Participant No. 9 from the pre-hospital emergency department said: *We have received less Karaneh (piecework wage) than the hospital staff and this has reduced the motivation of the guys. That is, it has reduced the incentive to cooperate and to work shift. We take all the traumatic patients to the hospital, and then, rumor has it that the earnings of triage staff are about twice as much as ours. It is 100% more*. Participant No.12 from hospital staff said: *The less the difference in the earnings and closer to reality, the less breach and the more cooperation would be*. Another participant said: *When you do a duty wholeheartedly and properly, then instead of being thanked for your effort, you're told 'you ruined that'. It drives us crazy at the very moment, but in the long run, it makes us indifferent. That is the worst possible scenario which should happen to someone, to me, he should quit his job.* The participants in the study considered lack of motivation as another challenging issue in collaboration between the two sectors and argued that the lack of attention to their motivational issues by managers would lead to their reluctance to cooperate.

### Low productivity of manpower.

Low productivity of manpower and not using human resources properly was another challenge for entering into a good collaboration between pre-hospital and hospital emergency staff. The use of low-less experienced and knowledgeable personnel had adverse impact on the process of collaboration and handover of the patients. Participant No. 9 said, *Sometimes, someone who takes delivery of a patient is a university student or someone who has newly come to triage and cannot take delivery of the patients properly. For example, he doesn't know the trauma mechanism well, but to the extent to sign the handover form and let us go.* Another participant from the pre-hospital emergency staff said, *I'm left with a sophomore student who is inexperienced and has not yet seen an accident scene.* Another participant in the pre-hospital emergency department said: *Anyone who works in a triage must have at least 5 years of work experience, but sometimes they are novice or students and cannot really understand the patient's real needs*. Lack of employing staff based on knowledge and experience not only creates some problems in the attainment of collaboration between pre-hospital and hospital staff, but it also affects the health outcomes of post-delivery of the patients. Poor knowledge and lack of experience of those who take delivery of the patients result in the loss of patients' information and consequently leads to inadequate care delivery.

### Non-common language

Not using a common language was one of the main categories to which all the participants in the study referred as a challenge in collaboration between the pre-hospital and in-hospital emergency wards. Lack of mutual understanding of each other's job as well as difference in training and experience were considered to be a factor in an ineffective cooperation.

### Difference in understanding and empathy.

The participants in this study believed that lack of mutual understanding and sympathy would diminish the cooperation in dealing with traumatic casualties. Participant No. 8 said: *The guys who have just worked in a pre-hospital emergency ward can never realize the present condition of the triage staff; similarly, many of the guys who have only worked in the triage can never understand the present condition of the one who has just removed the casualty of the accident scene*. Participant No. 3 said, *Nurses and emergency staff take guard. The hospital nurse wants to say that the emergency department does not do the job well, and the emergency technician says the hospital nurse does not do the work well and put the whole blame on others*. Participant No. 9 said: *The triage personnel are highly assertive. They think that their level of expertise and experience is much higher than the emergency staff, but we don’t think so. They think that we are on a lower scientific level and that’s why cooperation is being disturbed*. Shortage of mutual trust also increased the handover problems of the patients. The majority of the participants had the idea that the lack of understanding each other's circumstances prevents the occurrence of desired cooperation between the personnel in the handover of the patients / casualties.

### Difference in knowledge and experience.

Differences in degrees and academic degrees as well as practical and clinical experience of staff were the other challenge for collaboration. Participant No. 7 from the Pre-Hospital Emergency Department said: *Some medical emergency personnel are medical emergency technicians and some are nurse aides. Sure, their little knowledge does not allow them to take delivery of the patients from the pre-hospital emergency and this is the source of the problem*. Participant No. 8 from the hospital said: *Some time ago, one of the novice personnel who had not started his Service Plan, was put on the triage. I had brought a patient off the scene with TIA (transient ischemic attack). What I am saying is important because I'm the one who has seen the accident scene. The triage personnel who paid the patient a visit said that the patient had no problem at all, with no muscle weakness at all*! Another participant said*: While we are doing the handover of the patient to the triage, as the one in charge of the triage has not studied what the mechanism of the incident is, and he doesn't understand what it is, he doesn't heed our talk and our report*. One of the emergency personnel said: *The old hospital personnel while paying the patient a visit asks for the report, but not the novice ones. They talk to the patient, but not making any eye contact with him/her and they don't remember what they were told about*. The participant considered having enough training and experience as necessary for the reduction of problems in attainment of collaboration and emphasized the employment of experienced personnel in these two settings. 

## Discussion

The analysis of the findings of this study reveals that three groups of challenging factors- at organizational, managerial and individual levels are involved in the attainment of cooperation in Iran. Shortage of infrastructure resources at the organizational level, efficiency and ability of the managers at the managerial level, and the difference in the level of experience and education at the individual level greatly contribute to the creation of cooperation challenges. Most participants in this study emphasized on the shortage of infrastructure resources as the most important challenging factor in cooperating in the handover of traffic casualties. To them, shortage of equipment, insufficiency of physical space and shortage of manpower undeniably have a negative effect on the cooperation between these two important parts of the health system. In this regard, the findings of other researchers are in support of those of the present study. According to the findings of this study, inadequate physical space was another collaboration challenge in the handover of traffic casualties resulting in overcrowding of the hospital emergency ward and poor collaboration in taking delivery of the patients. 

To Trzeciak study,(13) overcrowding of the emergency ward is a very complicated issue whose adverse impact was emphasized in the ambulance delay in the hospital and a negative factor in the treatment process of casualties. Because in many cases the physical space of hospital emergency ward is sufficient enough to only accommodate injured patients under normal conditions,(14) it has contributed to the problem of transporting the casualties from pre-hospital emergency to the in-hospital emergency especially in urban areas.(15) Emergency room crowding is one of points of weakness in the country’s health system in consequences of which there are delayed ambulances and the hospital's lack of readiness to deal with crises.(13) Inadequate working physical space, emergency room crowding due to the prolongation of patient handover time and the delay of the emergency technicians were identified as one of the challenges of the health system in collaboration with these two systems. Another finding of the study was the shortage of manpower which directly had a negative effect on the cooperation of pre-hospital and in-hospital emergency staff in the handover of casualties. This finding is similar to that of Kralewski which emphasized the negative role of shortage of manpower on inter-ward cooperation.(16,17) Orthner *et al*.(18) study provides additional support for the shortage of emergency technicians as a factor in incomplete collection and recording of patient information from the accident scene and on the way to the hospital having adverse impact on collaboration. 

Inefficient management was considered as one of the main categories in the challenges of collaboration between the emergency department and the hospital in this study. This category was characterized by shaky accountability, doing activities on out-of-date basis or routine activity, poor motivation and low productivity of manpower. Vaismoradi et al believe that the delivery of safe care requires the cooperation of all health system staff which is attained through a capable and competent management. Mismanagement can lead to dangerous nursing practices.(19,20) Cooperation in delivery of health services and patient care under inept management resulting from poor accountability, unscientific health services and demotivated personnel meets undeniable challenges. 

Doing activities on out-of-date information basis or routinism was characterized by not using new scientific achievements in the handover process of the patient and insisting on taking therapeutic measures in the same routine from the past. Evans et al considered one of the delivery challenges as doing activities in the same routine and resistance to the use of advances in sciences and new technology.(21) Routinism was one of the causes of the scientific stagnation of a number of personnel which revealed itself in resistance to changes and promotion of scientific and practical knowledge. This challenge made a difference in the viewpoints of the staff who were willing to exploit new scientific information in the delivery of patient care and those who got used to doing activities on out-of-date basis was a barrier to desired collaboration.

Poor motivation management or not paying adequate attention to motivational issues in two material and spiritual dimensions was the other cooperation challenge. Discouragement, disillusionment, and lack of interest in collaboration resulted from poor motivation of the personnel. Smith and Rogers believed that paying attention to motivational issues and fairly giving points to activities in team working is very important.(22) To Bresnen and Marshall, "the use of incentives in partnering and alliancing has been seen as an important way of reinforcing collaboration in the short term and helping to build trust between clients and contractors in the long term".(23) In view of Vaismoradi et al, health service managers should pay attention to the encouragement of the personnel(20) because positive and negative incentives can be effective in helping individuals and groups work together to achieve collaboration.(23) When the authorities in the two systems disregard the motivational issues of the staff, their inclination and willingness to cooperate will peter out over time and it changes into a challenge in the handover of the patients. Another collaboration challenge was the low productivity of manpower with inefficient and inexperienced personnel in attendance to shifts which had an adverse impact on the process of cooperation. In a review study by Bost *et al*.,(24) knowledge, experience, and competency of the personnel were mentioned as important contributing factors in achieving collaboration. In addition, Owen *et al*.(25) considered staff's poor knowledge as one of the reasons for the development of handover problems as well as collaboration challenges. 

Lack of common language was another main category that overshadowed the collaboration of both pre-hospital and in-hospital emergency staff. 

Difference in understanding and empathy was the other cooperation challenge between pre-hospital and in-hospital emergency. The findings of this study are similar to the findings of a number of other studies. In Owen et al study, for instance, the difference in the description of staff's roles and responsibilities and work environment were mentioned as a challenging cooperation factor in the handover of the patients from ambulance personnel to hospital especially in critical situations. Boost *et al.*(25) in a review study, noted mutual understanding and sharing of skills and competencies as important cooperation factors.(24) Das and Teng(26) believed that the role of trust in the implications of the cooperation is firmly established. Trust demonstrates professional qualification and competency of a co-worker to fulfill a commitment, and reduces the dangers of inappropriate performance by other co-workers. In Iran, such factors as poor inter-group communication, distrust, and lack of awareness toward group work processes were reported to be a few collaborative challenges.(27) One of the important components of collaboration in any organization is the common language without which weakening of intra and extra-group communication and inability to share information desirably will make cooperation run up against a serious challenge.

Difference in knowledge and experience was another collaborative challenges based on the findings of the current study. Behara *et al.*(28) believe that as two organizations with different backgrounds and specialties are involved in the handover of patients from ambulance personnel to the hospital, cooperation can face challenges; therefore, it is necessary to hold interdisciplinary and multi-purpose training classes to promote collaboration and achieve a safe delivery of the patients. These trainings would increase mutual understanding and team work culture. Furthermore, the same triage procedure in both emergency and hospital leads to improvement of cooperation and handover quality.(29) In view of Bruce et al, the conversion of specialized vocational training to joint inter-professional training and doing teamwork will reduce the likelihood of the occurrence of injury at the time of patients' handover.(30) In addition, mutual awareness of roles and responsibilities and sharing skills and capabilities are considered among the key factors in achieving cooperation.(24)

## Conclusion

The results of this study have deepened the understanding of the challenges of co-operation between the two systems in road traffic accidents. In this study, confusion and delay of the participants in the handover the patients were attributed to the challenge of inadequate infrastructure resources. However, adequate provision of infrastructure resources such as equipment, physical space and manpower would lead into desired cooperation. Another challenge on the way of cooperation between these two systems is unscientific and ineffective management governing hospital emergency department with such components as shaky accountability, doing activities on out-of-date or traditional basis, poor motivation, and low productivity of manpower interferes with effective cooperation between pre-hospital and hospital emergency in the handover of road accident casualties. Scientific improvement of the handover process of road accident casualties and training the personnel of these two systems, as well as raising their knowledge and skills will maximize cooperation and minimize the problems. By exploiting the results of this study in careful planning of resources, promotion of managerial practices as well as empowerment program of the staff, healthcare managers and policymakers can take a pace forward in order to enter into a hearty coordination between these two systems.
